# Vascular Smooth Muscle Cell-Derived Exosomal MicroRNAs Regulate Endothelial Cell Migration Under PDGF Stimulation

**DOI:** 10.3390/cells9030639

**Published:** 2020-03-06

**Authors:** Jeongyeon Heo, Hee Cheol Yang, Won Jong Rhee, Hara Kang

**Affiliations:** 1 Department of Life Sciences, Incheon National University, Incheon 22012, Korea; godhjy409@nate.com; 2 Department of Bioengineering and Nano-Bioengineering, Incheon National University, Incheon 22012, Korea; aaaa428a@gmail.com (H.C.Y.); wjrhee@inu.ac.kr (W.J.R.); 3 Division of Bioengineering, Incheon National University, Incheon 22012, Korea; 4 Institute for New Drug Development, Incheon National University, Incheon 22012, Korea; 5 Division of Life Sciences, College of Life Sciences and Bioengineering, Incheon National University, Incheon 22012, Korea

**Keywords:** vascular smooth muscle cells, endothelial cells, intercellular communication, exosome, microRNA

## Abstract

Intercellular communication between vascular smooth muscle cells (VSMCs) and endothelial cells (ECs) is essential for the maintenance of vascular homeostasis. The presence of exosomes, a recently discovered player in vascular cell communication, has been associated with vascular disease progression. However, the detailed mechanism of how the signal mediated by exosomes affects the function of vascular cells during vascular pathogenesis is yet to be further understood. In this study, we investigated the expression of exosomal microRNAs (miRNAs) secreted by VSMCs and their functional relevance to ECs in pathogenesis, including their role in processes such as platelet-derived growth factor (PDGF) stimulation. We observed that PDGF stimulation contributes to a change in exosomal miRNA release from VSMCs; specifically, miR-1246, miR-182, and miR-486 were deficient in exosomes derived from PDGF-stimulated VSMCs. The reduced miRNA expression in these exosomes is associated with an increase in EC migration. These findings increase our understanding of exosome-mediated crosstalk between vascular cells under a pathological condition.

## 1. Introduction

Arterial injury stimulates the production of growth factors, such as platelet-derived growth factor (PDGF) by platelets and a variety of cellular elements associated with the vessel wall. Growth factor production induces a phenotypic change in vascular smooth muscle cells (VSMC) through a conversion of their state from a quiescent, “contractile”, to a proliferative, “synthetic”. This change is characterized by cell proliferation, migration, and secretion of matrix [1]. PDGF-induced phenotypic modulation contributes to the maintenance of vascular homeostasis. However, aberrant PDGF signaling is implicated in vascular remodeling, a complex pathological process of the vascular system, and contributes to a range of vascular pathologies including restenosis, atherosclerosis, and pulmonary hypertension [2,3]. This pathogenesis is characterized by dysfunction in major aspects of vasculature regulation, including abnormal proliferation, migration and apoptosis of endothelial cells (EC) and VSMCs. As intercellular communication is essential for the maintenance of tissue homeostasis and disease prevention, understanding the means of interaction between PDGF-modulated VSMCs and ECs might provide important insight into the development of vascular pathogenesis.

At short distances, cellular crosstalk can occur through direct cell-to-cell contact, while long-range communication can be mediated by cytokines or hormones. Recently, exosomes have emerged as a newly discovered route of communication among cells [4]. Exosomes are small, membrane-bound vesicles of different sizes, composition, and subcellular origin secreted by most cell types [5]. Exosomes contain various functional proteins, mRNAs and microRNAs (miRNA) [6]. The transfer of exosome contents to recipient cells has been described in a variety of cells; through cargo transfer to target cells, they act as regulators of intercellular communication between adjacent and remote cells [6]. The vesicle composition and biological content signify specific cellular states [7]. Moreover, microenvironmental stimuli can influence the quantity and variety of cargo transferred by exosomes through signals that induce biomolecule enrichment or depletion. [8]. Therefore, their potential use as diagnostic, prognostic and therapeutic markers in both physiological and pathological processes has raised significant interest [9,10].

In vascular homeostasis and cardiovascular disease progression, the role of exosomes as crucial regulators has been highlighted by recent investigations [10,11,12]. Exosome trafficking for the purpose of intercellular communication is suspected to contribute to vascular pathologies. Evidence shows that exosomes from mice with pulmonary hypertension are able to induce pulmonary hypertension in healthy mice by stimulating pulmonary vascular remodeling and right ventricular hypertrophy [13]. In addition, it has been discovered that exosomes derived from human pulmonary artery endothelial cells (PAEC) interact with pulmonary artery smooth muscle cells (PASMC) to induce proliferation [14], whereas exosomes derived from PASMCs influence migration and angiogenesis of PAECs [15].

Previous studies have illustrated the presence of miRNAs in circulation that act as a type of signaling molecule involved in intercellular communication [10]. Furthermore, the use of circulating miRNAs as biomarkers of cardiovascular diseases has been reported [16]. MiRNAs packaged in exosomes (exosomal miRNAs) can also influence the fate or function of receiving cells. For example, miRNAs secreted by glioma stem cells regulate the gene expression of receiving neural stem cells, particularly targeting genes involved in cell fate and tumorigenesis [17]. Although exosomes elicit functional effects on cells involved in vascular cell communication, little is known on the expression pattern and functional relevance of exosomal miRNA derived from VSMCs. Therefore, we aimed to investigate whether exosomal miRNAs secreted by VSMCs could act as a messenger implicated in EC processes of vascular homeostasis and pathogenesis.

## 2. Materials and Methods

### 2.1. Cell Culture

Human primary pulmonary artery smooth muscle cells (PASMCs) and human primary pulmonary artery endothelial cells (PAECs) were purchased from Lonza (CC-2581 and CC-2530) and were maintained in Sm-GM2 medium (Lonza, CC-3182) containing 5% fetal bovine serum (FBS) and EGM2 medium (Lonza, CC-3162) containing 2% FBS, respectively. For the production of exosomes from PASMCs, cells were cultured in media that contained exosome-free FBS. The exosome-free FBS was prepared by ultracentrifugation at 4 °C at 120,000 *g* for 10 h using a TLA-100.3 fixed angle rotor in an ultracentrifuge (Optima TL-100, Beckman Coulter, Brea, CA, USA). The supernatant was filtered using a 0.2-μm syringe-filter and stored at −20 °C. Recombinant human PDGF-BB was purchased from R&D Systems (220-BB). The cells were treated with 40 ng/mL PDGF-BB under starvation conditions. For starvation conditions, cells were maintained in Dulbecco’s modified egles medium (DMEM, SH30243.01) containing 0.2% FBS for 16 h.

### 2.2. Exosome Isolation

Culture medium was collected and exosomes were isolated using ExoQuick-TC^TM^ (System Biosciences, Palo Alto, CA, USA, EXOTC50A-1) according to the manufacturer’s instructions. Briefly, the medium was centrifuged at 3000 g for 15 min and the supernatant was incubated with the exosome precipitation solution at 4 °C overnight. After subsequent centrifugation at 1500 *g* for 30 min, the pellet was resuspended in phosphate buffered saline (PBS). Size distribution of the isolated exosomes was analyzed by nanoparticle tracking analysis (NTA) using NanoSight NS300 (Malvern Panalytical, Malvern, UK). BCA protein assay kit (Thermo Fisher Scientific, 23227, Waltham, MA, USA) was used for quantification of exosomes.

### 2.3. Quantitative Reverse Transcriptase-PCR (qRT-PCR)

For quantification of mature miRNAs, such as miR-182, miR-486 and miR-1246, the miScript PCR assay kit (Qiagen, MS00008855, MS00004284 and MS00043491, Hilden, Germany) was used according to the manufacturer’s instructions. Data analysis was performed using a comparative C_T_ method in the Bio-Rad software. MiRNA levels were normalized to U6 small nuclear RNA. The average of three experiments, each performed in triplicate, is presented with standard errors.

### 2.4. MiRNA mimics and anti-miRNA oligonucleotides

Chemically modified double-stranded RNAs designed to mimic the endogenous mature miR-182 (5′-UUUGGCAAUGGUAGAACUCACACU-3′), miR-486 (5′-UCCUGUACUGAGCUGCCCCGAG-3′) and miR-1246 (5′-AAUGGAUUUUUGGAGCAGG-3′) were purchased from Genolution (Seoul, Korea). Antisense inhibitor RNAs (anti-miR-182, anti-miR-486 and anti-miR-1246) and negative control miRNA were purchased from Bioneer (Daejeon, Korea) (anti-SMI-002 and SMC-2101). The miRNA mimics and anti-miRNA oligonucleotides were transfected at 5 nM and 50 nM, respectively, using RNAi Max (Invitrogen, 13778150, Carlsbad, California, CA, USA) or G-Fectin (Genolution) according to the manufacturer’s protocol.

### 2.5. Immunoblotting

Cells were lysed in TNE buffer (50 mM Tris–HCl (pH 7.4). 100 mM NaCl. 0.1 mM EDTA) and total cell lysates were separated by SDS-PAGE, transferred to PVDF membranes, immunoblotted with antibodies and visualized using an enhanced chemiluminescence detection system (Bio-Rad, Hercules, CA, USA). The antibodies used for immunoblotting were an anti-CD9 (EXOAB-CD9A-1), an anti-CD63 (EXOAB-CD63A-1), an anti-CD81 (EXOAB-CD81A-1), and an anti-HSP70 (EXOAB-HSP70A-1) from System Biosciences (Palo Alto, CA, USA).

### 2.6. Cell Proliferation Assay

CellTiter-Glo^®^ Luminescent Cell Viability Assay (Promega, G7572, Madison, WI, USA) was used to determine the number of viable cells in culture. Briefly, 5 × 10^3^ cells/well were seeded in 96-well plates in triplicate. After treatment with PDGF-BB or exosomes for 3 days, a volume of CellTiter-Glo reagent equal to the volume of cell culture medium was added to each well. The plates were shaken for 2 min to induce cell lysis and further incubated for 10 min to stabilize luminescent signal. As there is a linear relationship between the luminescent signal and the number of cells, cell proliferation was measured by reading the absorbance at 490 nm using a GloMax 96 Microplate Luminometer (Promega, Madison, WI, USA). Fold change was calculated as the ratio of recorded luminescence values.

### 2.7. In Vitro Scratch Wound Assay

PASMCs transfected with indicated miRNAs or treated with exosomes were plated in 6-well plates and three scratch wounds were generated with a 200 μL disposable pipette tip. Scratch wounds were photographed over 16 h with a Nikon inverted microscope (Nikon, Tokyo, Japan) with an attached digital camera and their widths were quantitated with ImageJ software. Distance of migration was calculated by subtracting the width measured at a given time from the width initially measured.

### 2.8. Next-Generation Sequencing (NGS)-Based Small RNA Sequencing

cDNA libraries were constructed with the small RNA library kit (NEB, Ipswich, MA, USA) using 3 μg of total RNA from PASMC-derived exosomes. To generate a library product, adapter ligation, reverse transcription, PCR amplification, and pooled gel purification were conducted. The RNA 3′-adapter is specifically modified to target miRNAs and other small RNAs that have a 3′-hydroxyl group resulting from enzymatic cleavage by Dicer or other RNA processing enzymes. The adapters are ligated to each end of the RNA molecule and an RT reaction is used to create single-stranded cDNA. The cDNA is then PCR amplified using a common primer and a primer containing one of 48 index sequences. The introduction of an index sequence at the PCR step separates the indexes from the RNA ligation reaction. To verify the size of the PCR enriched fragments, the template size distribution was checked by running on an Agilent Technologies 2100 Bioanalyzer (Santa Clara, CA, USA) using a DNA 1000 chip. The prepared libraries were quantified using qPCR according to the Illumina qPCR quantification protocol guide. Then, 50-nt single-end sequencing was performed by Illumina HiSeq2000 (Illumina, San Diego, CA, USA)

After removal of the adaptor sequence in the reads, all reads were clustered to find unique sequences and those were then counted. In order to identify miRNA sequences, the unique sequences found after clustering were searched on the miRBase database by blast. Trimmed reads were considered as miRNA on the condition that they possess 100% sequence identity.

MiRNAs with at least one zeroed read count value across all samples were excluded from the analysis. We added 1 to each count value to facilitate log2 transformation. Filtered data were logarithm-transformed and normalized by the Trimmed Mean of M-values (TMM) method.

### 2.9. Statistical Analysis

For each of the assays, three experiments were performed in triplicate, and the results were presented as the average with standard error. Statistical analyses were performed by an analysis of variance followed by Student’s *t* test using Prism 8 software (GraphPAD Software Inc., San Diego, CA, USA). *P* values of < 0.05 were considered significant and are indicated with asterisks.

## 3. Results

### 3.1. PASMC-Derived Exosomes Affect PAEC Migration under PDGF Stimulation

**Figure 1:**
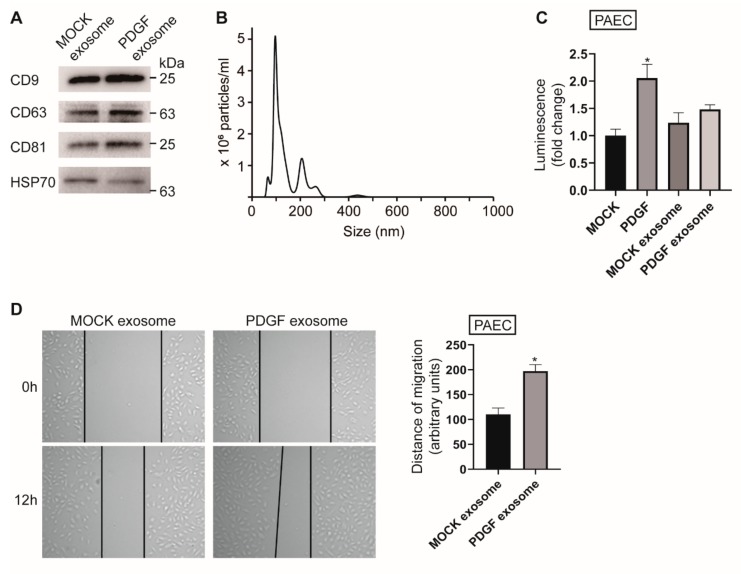
Exosomes secreted by platelet-derived growth factor (PDGF)-stimulated pulmonary artery smooth muscle cells (PASMCs) promote pulmonary artery endothelial cell (PAEC) migration. (**A**). Immunoblot analysis of exosomal protein markers, including CD9, CD63, CD81, and Hsp70, using exosomes isolated from PASMCs not treated with PDGF (MOCK exosome) and treated cells (PDGF exosome) (**B**). Size distributions and concentrations of exosomes analyzed by nanoparticle tracking analysis (NTA) (**C**). Luminescent signals for viable PAECs treated with PDGF-BB, MOCK exosome, or PDGF exosome for 3 days. * *p* < 0.05. (**D**). PAECs treated with MOCK exosome or PDGF exosome were subjected to the scratch wound assay. The distance of the migration was measured using ImageJ at 12 h after a scratch wound was introduced. The left panel shows representative images, and the right panel shows the quantification graph of the relative migration distance. The means ± S.E. of triplicate measurements of three independent experiments are shown; * *p* < 0.05.

As phenotypic modulation of VSMC is essential for the maintenance of vascular homeostasis and pathogenesis, we hypothesized that the signaling effects generated by exosomes secreted by VSMCs under PDGF stimulation might differ from those exerted by exosomes derived from untreated cells. To investigate whether PASMC-derived exosomes exposed to a PDGF signal play a regulatory role in the modulation of PAECs, we isolated exosomes from a PASMC medium following 40 ng/mL PDGF-BB stimulation. As exosomes are enriched with the tetraspanins, such as CD9, CD63, and CD81, we first analyzed the isolated exosomes by Western blot analysis of well-characterized exosomal protein markers including CD9, CD63, CD81, and Hsp70. All exosomal markers were detected in the exosomes isolated from the media of PASMCs under normal (MOCK exosome) and PDGF-stimulated (PDGF exosome) conditions (Figure 1A). In addition, the size of PASMC-derived vesicles was observed by nanoparticle tracking analysis [18]. The analysis showed that most exosomes exhibited a diameter of approximately 100 nm (Figure 1B).

As the proliferation of ECs is enhanced in PDGF-stimulated conditions, we examined whether exosomes secreted by PASMCs in PDGF-stimulated conditions exert an effect on the proliferation of ECs. PAECs were treated with 40 ng/mL PDGF-BB (PDGF) or 5 µg of exosomes derived from PASMCs, under normal (MOCK exosome) or PDGF-BB-stimulated conditions (PDGF exosome), for 72 h and subjected to a cell proliferation assay using CellTiter-Glo^®^ Luminescent Cell Viability Assay (Figure 1C). The concentration of exosomes used in this study was chosen based on a previous study [19]. As expected, the proliferation of ECs was enhanced by PDGF stimulation compared to the control condition (MOCK). PAECs treated with exosomes secreted by PASMCs under physiological conditions (MOCK exosome) did not have a significant effect on cell proliferation ability in comparison to the control PAEC cells (MOCK). PAECs treated with exosomes secreted by PASMCs in PDGF-stimulated conditions (PDGF exosome) did not affect the proliferation of ECs significantly compared to PAECs treated with MOCK exosome. These results suggest that PASMC-derived exosomes might not play a significant role in regulation of PAEC proliferation.

We subsequently examined whether exosomes secreted by PASMCs have functional effects on the migratory activity of PAECs. PAECs were treated with 5 µg of PASMC-derived exosomes and subjected to an in vitro scratch wound assay. We observed that migration of PAEC treated with PDGF exosome was enhanced compared to the control, which was PAEC treated with MOCK exosome (Figure 1D). This finding indicates that exosomes produced by PDGF-stimulated PASMCs affect PAEC migration and demonstrates that exosomes mediate the functional communication of PASMCs with PAECs under PDGF-stimulated pathogenic conditions. PASMCs might pack specific cargo molecules into exosomes or eliminate specific cargo molecules from exosomes in response to PDGF signal, for transfer into PAECs, which results in enhanced migratory activity of PAECs.

### 3.2. Exosomal miRNA Profile is Regulated by PDGF Signaling

**Figure 2:**
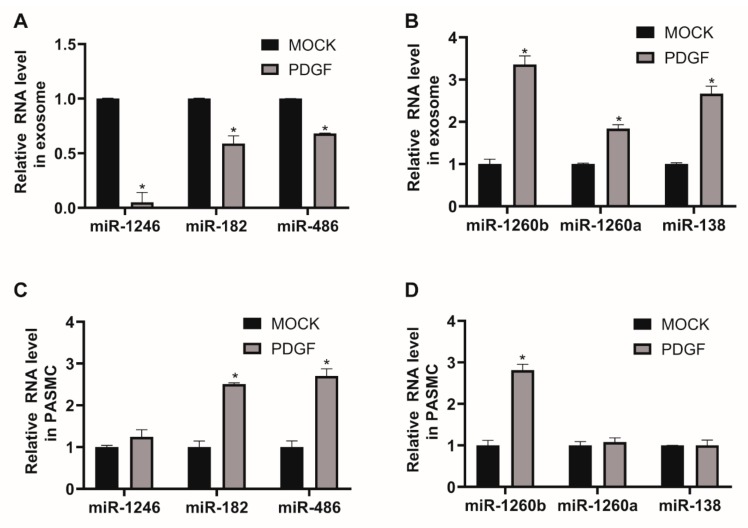
Expression of miRNAs in exosomes secreted by PASMCs is affected by PDGF stimulation. (**A**) and (**B**). Levels of miRNAs relative to U6 snRNA measured by qRT-PCR in exosomes secreted by PASMCs 24 h after exposure to PDGF-BB. Data represent the means ± S.E. of triplicates. * *p* < 0.05. (**C**) and (**D**). Levels of miRNAs relative to U6 snRNA measured by qRT-PCR in PASMCs 24 h after exposure to PDGF-BB. Data represent the means ± S.E. of triplicates. * *p* < 0.05.

Even though exosomal miRNAs are known to act as messengers of intercellular communication, specific miRNA expression patterns in exosomes from PDGF-stimulated VSMCs remain unknown. Thus, we investigated the miRNA profiles of PASMC-derived exosomes and their changes in response to a PDGF-BB signal using next-generation sequencing (NGS)-based small RNA sequencing (GSE145508). A library of small RNA sequencing was made of the total RNA extracted from isolated exosomes and one sample per condition was sequenced. We identified 41 downregulated miRNAs and 54 miRNAs upregulated by ≥ 2-fold in PASMC-derived exosomes, following PDGF signaling, in comparison to exosomes under normal conditions (Table 1). We validated the expression levels of certain down- or upregulated miRNAs from exosomes isolated under control or PDGF-stimulated conditions by qRT-PCR. Six highly expressed miRNAs in MOCK exosomes were selected. The levels of miR-1246, miR-182, and miR-486 decreased following PDGF signaling (Figure 2A), whereas the levels of miR-1260b, miR-1260a, and miR-138 in exosomes increased upon PDGF signaling (Figure 2B), which is consistent with the outcome of NGS-based small RNA sequencing.

We subsequently examined the cellular expression levels of miRNAs regulated by PDGF stimulation to determine whether the changes in exosomal miRNA levels depend on PDGF-induced regulation of the cells of origin, as exosomes contain molecular constituents of these cells (Figure 2C,D). The qRT-PCR analysis indicates that cellular levels of miR-1260b, which was enriched in PDGF-stimulated exosomes, was upregulated by PDGF-BB. However, endogenous levels of miR-1246, miR-1260a, and miR-138, which were tightly regulated miRNAs in PDGF-stimulated exosomes, were not changed by PDGF stimulation in PASMCs. Moreover, we observed that cellular levels of exosomal miRNAs downregulated by PDGF signaling, such as miR-182 and miR-486, were rather increased in response to PDGF stimulation, which suggests that miRNAs might have differential expression profiles depending on PDGF stimulation. These results highlight the importance of understanding the role of environmental stimuli on exosomal miRNA expression for vascular cell communications.

### 3.3. PASMC-Derived Exosomes Affect Cellular Levels of miRNA in PAECs

**Figure 3:**
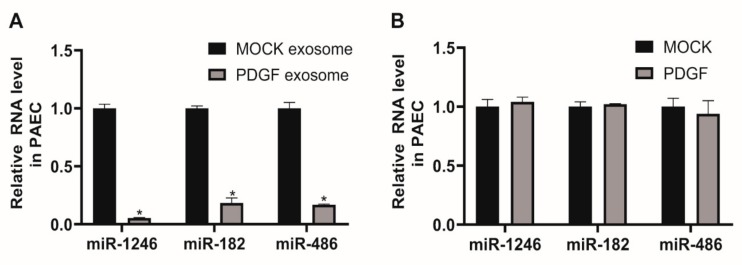
Levels of miR-1246, miR-182, and miR-486 in PAECs are affected by treatment with exosomes derived from PDGF-stimulated PASMCs. **A**. Levels of miRNAs relative to U6 snRNA measured by qRT-PCR in PAECs 24 h after treatment with PASMC-derived MOCK exosomes or PDGF exosomes. Data represent the means ± S.E. of triplicates. * *p* < 0.05. **B**. Levels of miRNAs relative to U6 snRNA measured by qRT-PCR in PAECs 24 h after exposure to PDGF-BB. Data represent the means ± S.E. of triplicates.

In this study, we focused on exosomal miRNAs downregulated in response to PDGF signaling, including miR-1246, miR-182, and miR-486, to investigate their cellular functions. We first determined whether downregulated exosomal miRNAs from PDGF-stimulated PASMCs affect cellular levels of miRNAs in PAECs. PAECs were treated with 5 μg of PASMC-derived MOCK exosomes or PDGF exosomes for 24 h. We then measured the levels of miR-1246, miR-182, and miR-486. The levels of these miRNAs in PAECs treated with PDGF exosomes were significantly lower than those in PAECs treated with MOCK exosomes (Figure 3A), reflecting a deficiency of miR-1246, miR-182, and miR-486 in PASMC-derived PDGF exosomes. Under PDGF-stimulated conditions, miR-1246, miR-182, and miR-486 might be transported less to PAECs through exosomes, in comparison to normal conditions, due to a deficiency in their concentration.

We subsequently determined whether cellular levels of miR-1246, miR-182, and miR-486 in PAECs are downregulated by PDGF signaling. PAEC were treated with PDGF-BB for 24 h and miRNA levels were measured by qRT-PCR (Figure 3B). Endogenous levels of miR-1246, miR-182, and miR-486 in PAECs were not significantly changed by PDGF stimulation, as compared to the untreated control (MOCK). This result further supports the idea that miRNAs in PASMC-derived exosomes merge into PAECs and affect cellular expression levels, which might result in changes in PAEC functions.

### 3.4. Downregulation of miR-1246, miR-182, and miR-486 Elicits a Pro-Migratory Effect in Vascular Cells

**Figure 4:**
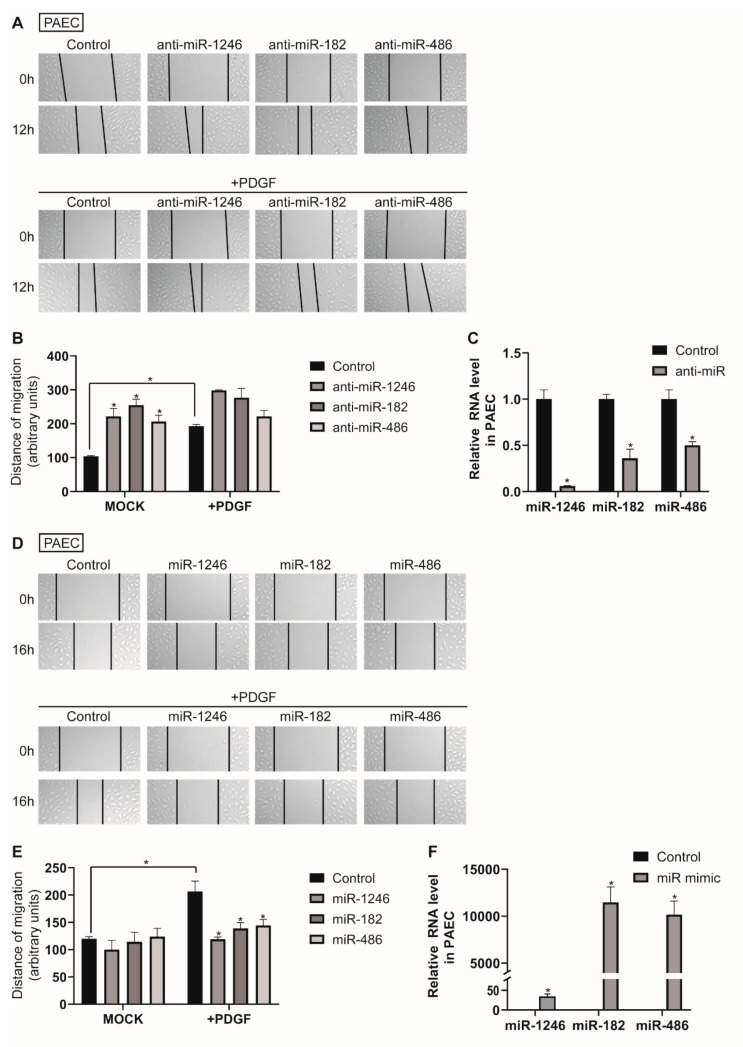
Downregulation of miR-1246, miR-182, and miR-486 promotes PAEC migration. (**A**). PAECs transfected with control or anti-miRNAs were subjected to the scratch wound assay in the presence or absence of PDGF-BB. The distance of the migration was measured using ImageJ at 12 h after a scratch wound was introduced. (**B**). The quantification graph of the relative migration distance. The means ± S.E. of triplicate measurements of three independent experiments are shown; * *p* < 0.05. (**C**). Levels of miRNAs relative to U6 snRNA measured by qRT-PCR in PAECs transfected with control or anti-miRs. Data represent the means ± S.E. of triplicates. * *p* < 0.05. (**D**). PAECs transfected with control or miRNA mimics were subjected to the scratch wound assay in the presence or absence of PDGF-BB. The distance of the migration was measured using ImageJ at 16 h after a scratch wound was introduced. (**E**). The quantification graph of the relative migration distance. The means ± S.E. of triplicate measurements of three independent experiments are shown; * *p* < 0.05. (**F**). Levels of miRNAs relative to U6 snRNA measured by qRT-PCR in PAECs transfected with control or miRNA mimics. Data represent the means ± S.E. of triplicates. * *p* < 0.05.

To investigate the cellular functions of exosomal miRNAs, we assessed the effect of antisense inhibitor RNAs (anti-miRNAs) and miRNA mimics on the migratory activity of PAECs. PAECs were transfected with anti-miRNAs against miR-1246, miR-182, or miR-486 for 48 h and subjected to an in vitro scratch wound assay (Figure 4A,B). Downregulation of miR-1246, miR-182, or miR-486 increased the migration rate of PAECs by approximately 2.1-, 2.4-, or 2-fold, respectively, compared with the negative control miRNA-transfected cells (Control), and further enhanced PAEC migration in PDGF-stimulated conditions, which implies the involvement of these miRNAs in PAEC migration. Anti-miRNA-mediated knockdown of miR-1246, miR-182, and miR-486 were confirmed by measuring miR-1246, miR-182, and miR-486 levels in PAECs at 48 h after transfection with anti-miRNAs via qRT-PCR (Figure 4C).

Subsequently, PAEC were transfected with miR-1246, miR-182, or miR-486 mimics for 24 h and subjected to an in vitro scratch wound assay (Figure 4D,E). We observed that the distance of migration was not significantly changed in cells transfected with miR-1246, miR-182, or miR-486 mimics as compared with the control mimic-transfected cells. Even if the target mRNA is suppressed by the exogenous miRNA mimics, it may not significantly reduce cell migration due to the functional existing target proteins. However, exogenous miR-1246, miR-182, or miR-486 abolished PDGF-induced migration of PAECs, which indicates that these miRNAs are sufficient to regulate PAEC migration under PDGF-stimulated conditions. Expression of exogenous miR-1246, miR-182, and miR-486 was confirmed by qRT-PCR (Figure 4F).

**Figure 5:**
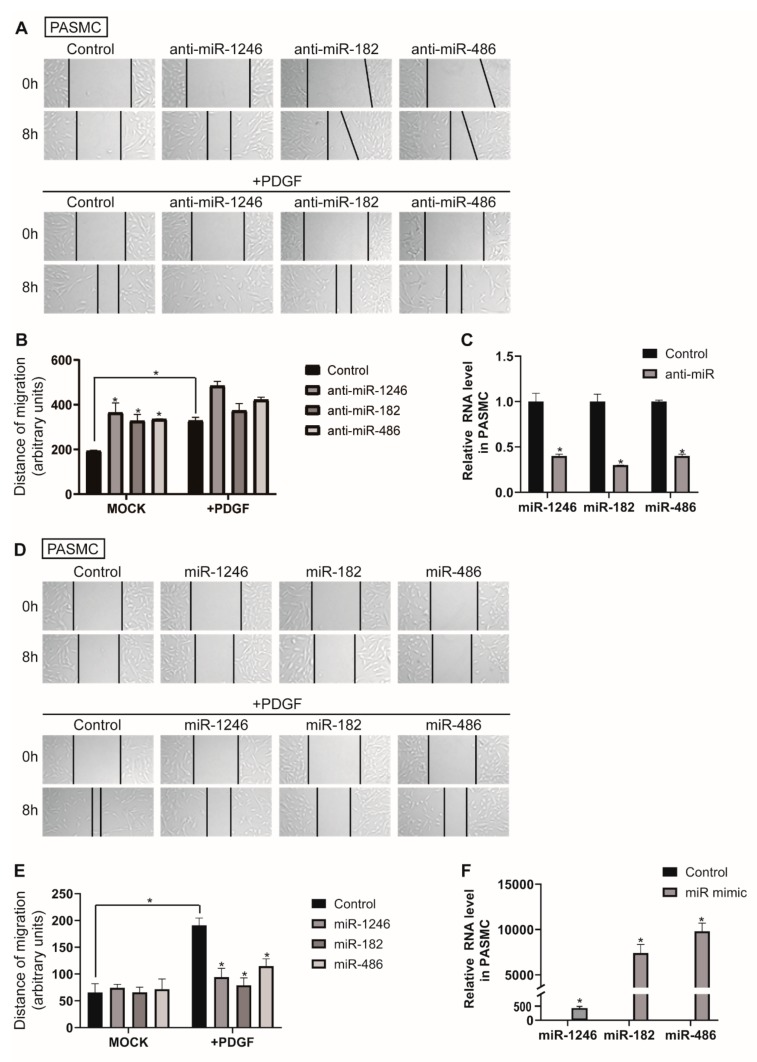
Downregulation of miR-1246, miR-182, and miR-486 promotes PASMC migration. (**A**). PASMCs transfected with control or anti-miRNAs were subjected to the scratch wound assay in presence or absence of PDGF-BB. The distance of the migration was measured using ImageJ at 8 h after a scratch wound was introduced. (**B**). The quantification graph of the relative migration distance. The means ± S.E. of triplicate measurements of three independent experiments are shown; * *p* < 0.05. (**C**). Levels of miRNAs relative to U6 snRNA measured by qRT-PCR in PASMCs transfected with control or anti-miRs. Data represent the means ± S.E. of triplicates. * *p* < 0.05. (**D**). PASMCs transfected with control or miRNA mimics were subjected to the scratch wound assay in the presence or absence of PDGF-BB. The distance of the migration was measured using ImageJ at 8 h after a scratch wound was introduced. (**E**). The quantification graph of the relative migration distance. The means ± S.E. of triplicate measurements of three independent experiments are shown; * *p* < 0.05. (**F**). Levels of miRNAs relative to U6 snRNA measured by qRT-PCR in PASMCs transfected with control or miRNA mimics. Data represent the means ± S.E. of triplicates. * *p* < 0.05.

We also explored whether miR-1246, miR-182, and miR-486 are involved in PASMC migration. The distance of cell migration was measured in PASMCs transfected with control or anti-miRNAs (Figure 5A,B). Migration of PASMCs was promoted when miR-1246, miR-182, or miR-486 was downregulated by approximately 1.9-, 1.7-, or 1.73-fold, respectively, compared with the negative control miRNA-transfected cells, and was further accelerated in PDGF-stimulated conditions. These results suggest that downregulation of miR-1246, miR-182, and miR-486 is critical for eliciting a pro-migratory effect. Knockdown of miR-1246, miR-182, and miR-486 was confirmed by qRT-PCR (Figure 5C). Subsequently, an in vitro scratch wound assay was performed with PASMCs transfected with either the control, miR-1246, miR-182, or miR-486 mimics (Figure 5D,E). Exogenous miR-1246, miR-182, and miR-486 did not significantly affect migration of PASMCs in the absence of PDGF stimulation; however, they significantly inhibited PDGF-induced migration of PASMCs. These results are consistent with the outcome of the in vitro scratch wound assay in PAECs. Therefore, miR-1246, miR-182, and miR-486 might be implicated in the regulation of vascular cell migration under PDGF stimulation. Levels of exogenous miR-1246, miR-182, and miR-486 were measured by qRT-PCR (Figure 5F).

### 3.5. Exosome-Derived miR-1246, miR-182, and miR-486 Counteract the Effect of Exosomes from PDGF-Stimulated PASMCs on PAEC Migration

**Figure 6:**
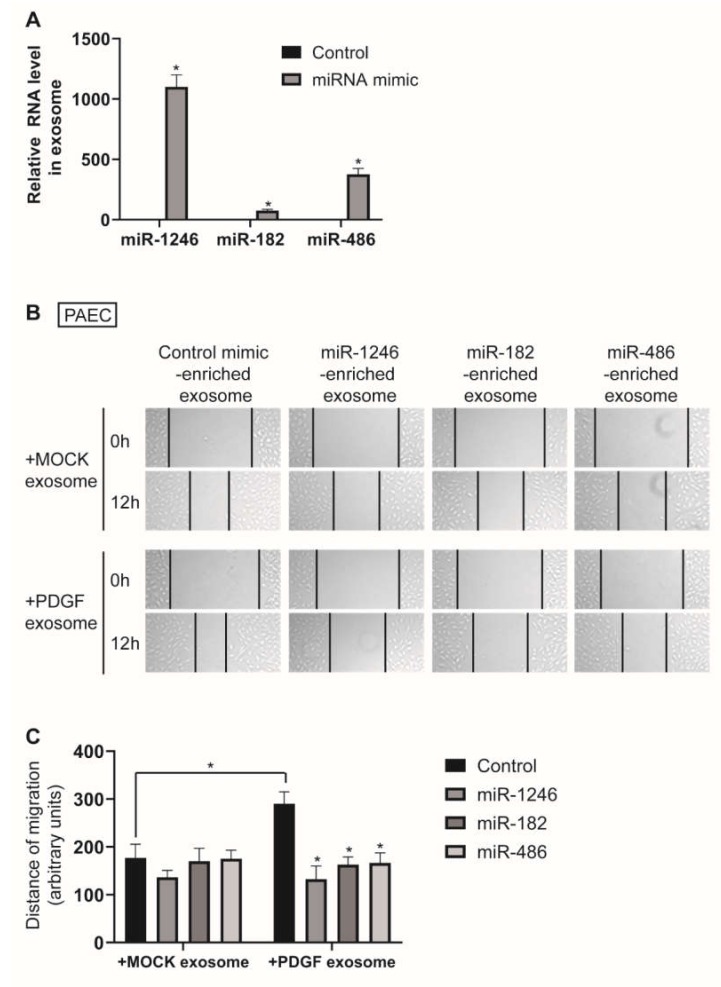
miR-1246, miR-182, or miR-486-enriched exosomes inhibit PAEC migration induced by exosomes from PDGF-stimulated PASMCs. (**A**). Levels of miRNAs relative to U6 snRNA measured by qRT-PCR in exosomes secreted by PASMCs transfected with indicated miRNAs. Data represent the means ± S.E. of triplicates. * *p* < 0.05. (**B**). PAECs treated with MOCK exosomes or PDGF exosomes were cotreated with indicated miRNA-enriched exosomes. Cells were then subjected to the scratch wound assay. The distance of the migration was measured using ImageJ at 12 h after a scratch wound was introduced. (**C**). The quantification graph of the relative migration distance. The means ± S.E. of triplicate measurements of three independent experiments are shown. * *p* < 0.05.

To investigate the function of exosome-derived miR-1246, miR-182, and miR-486, we isolated miR-1246-, miR-182-, or miR-486-enriched exosomes from the medium of PASMCs transfected with miR-1246, miR-182, or miR-486 mimics for 24 h. Enrichment of miR-1246, miR-182, and miR-486 in the exosomes was confirmed by qRT-PCR (Figure 6A). PAECs were treated with either MOCK exosomes or PDGF exosomes together with miR-1246-, miR-182-, or miR-486-enriched exosomes and subjected to an in vitro scratch wound assay. We observed that the pro-migratory effect of PDGF exosomes on PAECs was abrogated by miR-1246-, miR-182-, or miR-486-enriched exosomes, which suggests that exosome-derived miR-1246, miR-182, and miR-485 prevent exosome-mediated communication between PASMCs and PAECs in response to a PDGF signal (Figure 6B,C). These results suggest that depletion of miR-1246, miR-182, and miR-486 in PASMC-derived exosomes is critical to the enhanced migratory phenotype of PAECs under PDGF stimulation in pathogenic conditions.

## 4. Discussion

Exosomes are known to be mediators of intercellular communication in vascular diseases, including atherosclerosis and pulmonary artery hypertension [9]. As phenotypic modulation of VSMCs in response to PDGF is a hallmark of vascular repair and remodeling, we hypothesized a potentially important physiological role for exosomes derived from PDGF-stimulated VSMCs [20]. In this study, we found that PAEC migration is enhanced by exosomes released by PDGF-stimulated PASMCs. As exosomes are known to be effective carriers of miRNAs, we compared miRNA expression patterns in exosomes from PASMCs under normal and PDGF-stimulated conditions by NGS-based small RNA sequencing and found that miR-1246, miR-182, and miR-486 were deficient in exosomes derived from PDGF-stimulated PASMCs. Due to significant differential expression of exosomal miRNAs, we hypothesized that changes in exosomal miRNA levels derived from PDGF-stimulated PASMCs affect the PAEC phenotype. Thus, we further explored the functional relationships between changes in the concentrations of exosomal miRNAs derived from PASMCs and the migration activities of PAECs. Indeed, downregulation of miR-1246, miR-182, and miR-486 in exosomes derived from VSMCs affects endothelial migratory activity. The differential exosomal miRNA expression identified in this study suggests a contribution of exosomal miRNA to vascular homeostasis and provides the experimental basis for further understanding of vascular pathogenesis.

Recent studies have suggested the importance of cell-to-cell transport of exosomes in vasculature [21]. Kapustin et al., reported that VSMCs secrete exosomes that promote vascular calcification in response to environmental calcium stress [18,22]. Deng et al., reported that miR-143-3p is abundant in PASMC-derived exosomes and proposed its involvement in the promotion of PAEC migration [15]. However, specific exosomal miRNA expression patterns from VSMCs remain largely unknown. In this study, we determined that the exosomal miRNA profile is altered in PDGF-stimulated PASMCs and we identified a novel exosome-mediated miRNA communication between PASMCs and PAECs contributing to endothelial cell migration.

An important role of miR-1246, miR-182, and miR-486 in the pathogenesis of various cancers has already been revealed. These miRNAs illustrate oncogenic or tumor-suppressive functions, depending on the type of cancer. MiR-1246 is known to promote growth and metastasis of colorectal cancer cells and hepatocellular carcinoma by targeting CCNG2 or cell adhesion molecule 1, while miR-1246 inhibits cell invasion and epithelial-mesenchymal transition in prostate cancer and lung cancer cells by inhibiting N-cadherin and vimentin activities or targeting CXCR4 [23,24,25,26]. MiR-182 shows oncogenic features by promoting growth in oral squamous cell carcinoma, hepatocellular carcinoma, gastric cancer cells, and bladder cancer cells through repressing CAMK2N1, FOXO3a, RAB27A, Smad4, or RECK, while miR-182 plays a role as a tumor suppressor in renal cell carcinoma, Ewing’s sarcoma, and fibromatosis [27,28,29,30,31]. MiR-486 is known to drive tumorigenesis by targeting multiple negative regulators of PTEN/PI3K/Akt, FOXO, and TGF-β/Smad2 signaling in cervical cancer and prostate cancer cells [32,33]. In contrast, miR-486 inhibits cell proliferation and invasion through repressing GAB2, PIK3R1, Snail, neuropilin-2, CDK4, or PIM-1 in non-small-cell lung cancer, colorectal cancer, prostate cancer, hepatocellular carcinoma, and breast cancer cells [34,35,36,37,38,39,40]. In addition to previous findings, this study sheds light on the anti-migratory function of miR-1246, miR-182, and miR-486 in vascular cells, VSMC, and EC.

To understand the exact mechanism by which the miR-1246, miR-182, and miR-486 affect cell migration, we attempted to identify the predicted common target mRNAs of the three miRNAs using target prediction algorithms, such as Targetscan, miRanda, miRWalk, and RNA22. After PAECs were transfected with each miRNA, expressions of six predicted target genes, Ral GEF With PH Domain And SH3 Binding Motif 2 (RALGPS2), Ubiquitin Associated And SH3 Domain Containing B (UBASH3B), Insulin Like Growth Factor 1 Receptor (IGF-1R), Arginyltransferase 1 (ATE1), Pseudopodium Enriched Atypical Kinase 1 (PEAK1), and Dishevelled Associated Activator Of Morphogenesis 1 (DAAM1) were measured by qRT-PCR (Supplementary Figure S1). Only DAAM1 expression was reduced by approximately 43% by miR-1246. Since we could not identify common targets for the three miRNAs, we are currently conducting experiments to identify the target genes for each of these miRNAs. Especially, we are searching for the target genes of these miRNAs involved in cellular adhesion or cytoskeleton rearrangement.

Exosomes deliver a variety of molecules to the recipient cells in normal and pathological conditions. Most studies have focused on exosome content that is enriched in certain cell states. There is limited research on signal transmission to target cells by removal of specific miRNAs from exosomes. We observed that specific miRNAs are enriched or deficient in exosomes secreted from PDGF-stimulated PASMCs. Thus, we investigated the effect of miRNAs that are not secreted via exosomes on the recipient cells in a pathological environment. Exosomes lacking specific miRNAs, such as miR-1246, miR-182, and miR-486, that inhibit cell migration, successfully promoted PAEC migration, while the reverse phenotype was observed when these miRNAs were enriched. These results suggest that exosomes secreted from VSMCs transfer miR-1246, miR-182, and miR-486 into ECs and inhibit their migratory activities in physiological conditions. Therefore our findings imply that exosomal miRNAs play an essential role in the maintenance of vascular homeostasis as well as the progression of pathologies.

Significant differences in miRNA profiles have often been observed between exosomes and cells of origin [41,42]. Moreover, the exosomal miRNA profiles from a single cell types can be significantly altered by environmental stimuli. For example, hypoxia can lead to a change in miRNA levels in exosomes [43]. In this study, we observed that the exosomal miRNA profile of VSMCs was altered in response to PDGF stimulation and the change did not reflect its state in the cell of origin. These results imply that the selective cargo-sorting mechanism and its tight regulation exist so that specific intracellular miRNAs are delivered into exosomes in response to specific environmental conditions. However, activation and modulation of exosome biogenesis are still largely unknown. To develop a novel therapeutic approach for enhancing vascular repair, the molecular mechanism of miRNA-selective packaging into exosomes under physiological and pathological conditions remains to be explored.

## Figures and Tables

**Table 1:** PASMC-derived exosomal miRNAs regulated by PDGF-BB signal from next-generation sequencing (NGS) analysis.

Downregulated miRNA	Fold Change (PDGF Exosome/MOCK Exosome)	Upregulated miRNA	Fold Change (PDGF Exosome/MOCK Exosome)
miR-1246	−13.33	miR-409	9.24
miR-195-3p	−4.35	miR-576-3p	4.77
miR-4682	−3.91	miR-874	4.42
miR-320d	−3.86	miR-92a-1	4.03
miR-4645-3p	−3.80	miR-4741	3.91
miR-6511a	−3.73	miR-561-3p	3.91
miR-6511b	−3.64	miR-1273d	3.73
miR-1290	−3.52	miR-340-3p	3.52
miR-5189	−3.52	miR-412	3.41
miR-103a-2	−3.43	miR-193b	3.35
miR-320c	−3.40	miR-5100	3.29
miR-3613	−3.37	miR-26a-2-3p	3.26
miR-3074	−3.13	miR-147b	3.13
miR-3653-3p	−3.13	let-7g-3p	3.09
miR-1270	−3.10	miR-190a	3.00
miR-1249-3p	−3.09	miR-33b-3p	2.92
miR-375	−3.09	miR-1271-3p	2.81
miR-4775	−2.98	miR-4638-3p	2.81
miR-5010-3p	−2.81	miR-188	2.80
miR-3913	−2.79	miR-2277-3p	2.75
miR-939	−2.74	miR-1260b	2.60
miR-548h	−2.69	miR-3173	2.56
miR-182	−2.68	miR-3911	2.56
miR-486	−2.56	miR-6500-3p	2.56
miR-302b-3p	−2.56	miR-30b-3p	2.53
miR-3605-3p	−2.56	miR-5000-3p	2.50
miR-3619-3p	−2.56	miR-4485-3p	2.47
miR-580-3p	−2.56	miR-1271	2.46
miR-579	−2.53	miR-501-3p	2.39
miR-4466	−2.44	miR-1260a	2.30
miR-4449	−2.44	miR-1972	2.27
miR-6723	−2.43	let-7c-3p	2.25
miR-1228	−2.40	miR-29b-2	2.25
miR-668-3p	−2.31	miR-627-3p	2.25
miR-362-3p	−2.27	miR-193a-3p	2.25
miR-664a-3p	−2.26	miR-1224	2.25
miR-643	−2.25	miR-1306-3p	2.20
miR-664b-3p	−2.25	miR-4745	2.20
miR-485-3p	−2.15	miR-4787	2.20
miR-1273f	−2.06	miR-487a	2.20
miR-374a-3p	−2.06	miR-769-3p	2.19
		miR-656	2.17
		miR-6886	2.17
		miR-138	2.13
		miR-214	2.10
		let-7f-1-3p	2.10
		miR-221	2.09
		miR-100	2.08
		miR-1908-3p	2.06
		miR-337	2.05
		miR-99b	2.03
		miR-4792	2.03
		miR-1287	2.01
		miR-4767	2.00

## Supplementary Materials

The following are available online at https://www.mdpi.com/2073-4409/9/3/639/s1, Figure S1: Levels of predicted target mRNAs in PAECs transfected with miRNA mimics.
