# Supplementary figures and images for: Vascular Smooth Muscle Cell-Derived Exosomal MicroRNAs Regulate Endothelial Cell Migration Under PDGF Stimulation
Source: Cells. 2020 Mar 6;9(3):639. doi: 10.3390/cells9030639 (PMC7140448; doi:10.3390/cells9030639)

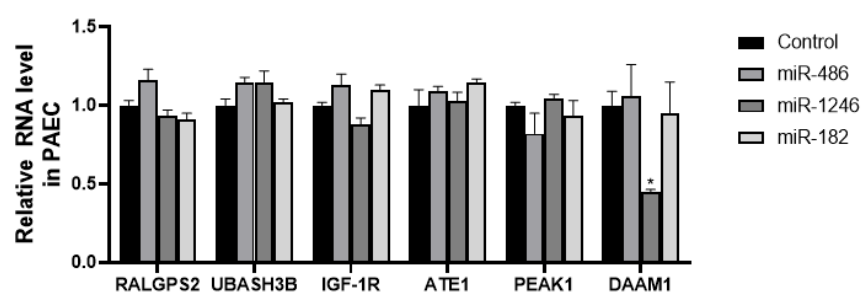

**Figure S1.** Levels of predicted target mRNAs in PAECs transfected with miRNA mimics.

Supplement: Supplementary file 1 [file cells-09-00639-s001.pdf]
